# Potential hazards associated with combustion of bio-derived versus petroleum-derived diesel fuel

**DOI:** 10.3109/10408444.2012.710194

**Published:** 2012-08-08

**Authors:** Jürgen Bünger, Jürgen Krahl, Olaf Schröder, Lasse Schmidt, Götz A. Westphal

**Affiliations:** 1 Institute for Prevention and Occupational Medicine of the German Social Accident Insurance, Institute of the Ruhr-University Bochum (IPA), Bochum, Germany; 2 Coburg University of Applied Sciences and Arts, Coburg, Germany; 3 Institute of Agricultural Technology and Biosystems Engineering, Johann Heinrich von Tünen Institute, Braunschweig, Germany

**Keywords:** Biodiesel, hydrotreated vegetable oil, diesel engine exhaust, exposure, health hazard

## Abstract

Fuels from renewable resources have gained worldwide interest due to limited fossil oil sources and the possible reduction of atmospheric greenhouse gas. One of these fuels is so called biodiesel produced from vegetable oil by transesterification into fatty acid methyl esters (FAME). To get a first insight into changes of health hazards from diesel engine emissions (DEE) by use of biodiesel scientific studies were reviewed which compared the combustion of FAME with common diesel fuel (DF) for legally regulated and non-regulated emissions as well as for toxic effects. A total number of 62 publications on chemical analyses of DEE and 18 toxicological *in vitro* studies were identified meeting the criteria. In addition, a very small number of human studies and animal experiments were available. In most studies, combustion of biodiesel reduces legally regulated emissions of carbon monoxide, hydrocarbons, and particulate matter. Nitrogen oxides are regularly increased. Among the non-regulated emissions aldehydes are increased, while polycyclic aromatic hydrocarbons are lowered. Most biological *in vitro* assays show a stronger cytotoxicity of biodiesel exhaust and the animal experiments reveal stronger irritant effects. Both findings are possibly caused by the higher content of nitrogen oxides and aldehydes in biodiesel exhaust. The lower content of PAH is reflected by a weaker mutagenicity compared to DF exhaust. However, recent studies show a very low mutagenicity of DF exhaust as well, probably caused by elimination of sulfur in present DF qualities and the use of new technology diesel engines. Combustion of vegetable oil (VO) in common diesel engines causes a strongly enhanced mutagenicity of the exhaust despite nearly unchanged regulated emissions. The newly developed fuel “hydrotreated vegetable oil” (HVO) seems to be promising. HVO has physical and chemical advantages compared to FAME. Preliminary results show lower regulated and non-regulated emissions and a decreased mutagenicity.

## Introduction

**Figure 1:**
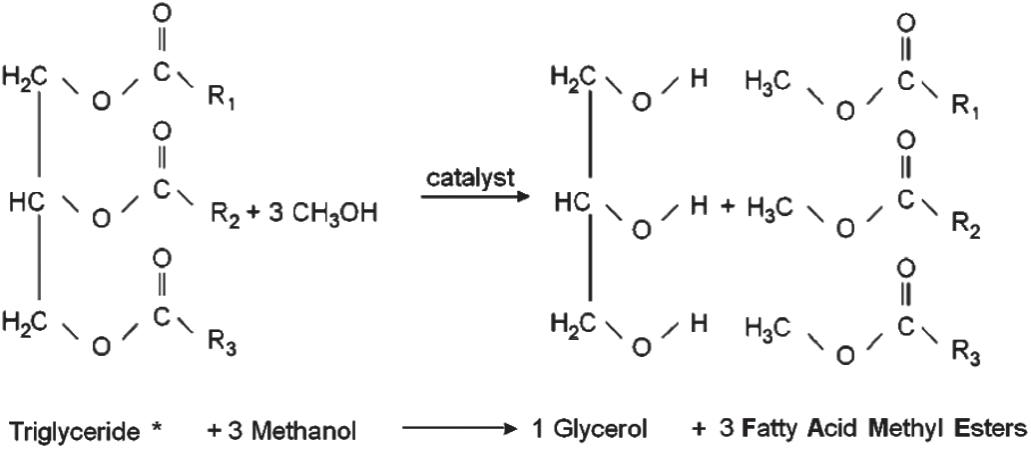
Scheme of biodiesel production by transesterification of triglycerides with methanol. Vegetable oils can be gained from different sources, e.g. rapeseed (canola), soybean, sunflower, palm fruit, coconut, animal fat.

The replacement of petroleum-derived fuels by biofuels from renewable resources has gained worldwide interest and is scientifically investigated for its environmental costs and benefits (Hill et al., 2006, Ragauskas et al., 2006). In particular, the reduction of atmospheric greenhouse gas was intensely discussed, since the combustion of bioderived fuels reduces net greenhouse gas emissions compared to fossil fuels (Koonin, 2006). Less attention has been paid to the possible hazards for human health (Krahl et al., 2001; Swanson et al., 2007). Bioethanol used in ignition engines and biodiesel for diesel engines are the most widely distributed renewable fuels worldwide. This review focuses on biodiesel. It is produced by transesterification of lipids (triglycerides) from vegetable oils with short chain alcohols, mainly methanol. Transesterification of triglycerides to the corresponding methyl esters proceeds by the reaction of triglyceride methanol and glycerol to 3 fatty acid methyl esters (FAME). Biodiesel can be produced from triglycerides of all kinds of oil plants, e.g. rapeseed (canola), palm oil, soybean, sunfower, jatropha, coconut, peanut, and even animal fat (Figure 1).

Biodiesel has similar properties as mineral oil-derived fuel and can be used in pure form (B100) or blended with common diesel fuel (DF) at any concentration (Knothe et al., 2010). Biodiesel use has been increased in the USA mainly as a 20% blend with DF (B20), since the “Energy Policy Act” came into force. ASTM D6751-08 details the US specifications for biodiesel blends. Biodiesel was introduced into the European market in the 1988s as B100. In 2003, fuel suppliers were committed to include increasing amounts of renewable fuels in all transport fuel sold in the EU by the Directive 2003/30/EC. At the same time, the European Committee for Standardization (CEN) released the standard EN 14214 for biodiesel which was updated in 2008. Currently, DF is supplemented with 7% biodiesel (B7) in Germany.

Diesel engine emissions (DEE) are highly complex mixtures. They consist of a wide range of organic and inorganic compounds which are distributed among the gaseous and particulate phases. Public health concern has arisen about DEE for these reasons (HEI, 1995). Most particles of DEE are nanoscaled making them readily respirable. These particles have hundreds of chemicals adsorbed onto their surfaces, including many known or suspected mutagens and carcinogens, e.g. polycyclic hydrocarbons (PAH) and nitrated polycyclic hydrocarbons (nPAH). The gaseous phase contains many irritants and toxic chemicals, e.g. aldehydes. Also nitrogen oxides (NO_*x*_), which are irritants and ozone precursors, are among the combustion products in the gaseous phase.

Exposures to DEE and their atmospheric transformation products occur often in both environmental and occupational settings. Since 1995, Health Effects Institute (HEI) has published a series of special reports and research papers – most of the reviews written by interdisciplinary expert panels – dealing with health effects of DEE (HEI, 1995, 1999, 2010). According to these reviews, there is suffcient evidence supporting a causal relationship between DEE and acute health effects, namely the exacerbation of asthma. The experts also found a suggestive evidence of a causal relationship with chronic health effects like childhood asthma, non-asthma respiratory symptoms, impaired lung function, total and cardiovascular mortality, and cardiovascular morbidity. A causal relationship of exposures to DEE and lung cancer was suggestive for occupational settings but not for the general population. US–EPA released a health assessment document concerning DEE in 2002. “The assessment concludes that long-term inhalation exposure is likely to pose a lung cancer hazard to humans, as well as damage the lung in other ways depending on exposure. Short-term exposures can cause irritation and inflammatory symptoms of transient nature. These effects seem to be highly variable across the population. The assessment also indicates that evidence for exacerbation of existing allergies and asthma symptoms is emerging” (EPA, 2002a).

Also in 2002, US–EPA released a Draft Technical Report on impacts of biodiesel on exhaust emissions (EPA, 2002b). The analysis mainly comprised data on regulated emissions, showing a reduction of 10.1% for particulate matter (PM), of 21.1% for hydrocarbons (HC), and 11.0% for carbon monoxide (CO) with the use of B20. Nitrogen oxides (NO_*x*_) increased by 2.0%. In addition, 11 “air toxics” were evaluated, including mainly small carbonyls and aromatics which are associated with the gaseous phase of the emissions. A small reduction of these compounds was calculated overall. McCormick (2007) included more recent data in a short review. He found the evaluation of US–EPA confirmed and added some studies providing results of biological assays showing a reduced mutagenicity.

In several critical reviews of the literature on health effects of DEE, Hesterberg and coworkers highlighted weaknesses and shortcomings of the studies on which HEI and EPA conclusions on DEE were based (Bunn et al., 2004; Hesterberg et al., 2005, 2006, 2009, 2010). Regarding the epidemiological studies concerning the lung cancer risk the lack of contemporaneous measurements of DEE exposures, uncertainties concerning exposure history, and inadequate consideration of confounding exposures such as gasoline exhaust and cigarette smoke were criticized (Bunn et al., 2004). Additionally the lacking dose response relationship was pronounced with regard to the observation that underground miners experiencing the highest exposures to DEE did not show elevations in lung cancer (Hesterberg et al., 2006). Moreover, animal studies showed lung tumors only in rats but not in mice or Syrian hamsters, and in rats only under “lung overload” conditions (Hesterberg et al., 2005). According to clinical and exposure studies showing lung inflammatory effects and thrombogenic and ischemic effects of inhaled DEE in humans it was remarked that unrealistically high DEE concentrations from older-model diesel engines were used (Hesterberg et al., 2009). Additionally, till now no mechanism of action was established allowing a reliable prediction of adverse health effects of DEE and it is uncertain which DEE constituents underlie the observed responses. Thus, Hesterberg and his colleagues emphasize the need for studies using realistic environmental and occupational exposures from new technology diesel engines including low sulfur fuels and exhaust after-treatment (Hesterberg et al., 2010).

In fact, during recent years strong efforts were made to minimize DEE-related health hazards. This includes improved combustion, exhaust after-treatment, the reduction of sulfur and aromatics, and the introduction of reformulated fuels. Therefore the contemporaneous risk assessment may be based on over-aged data. Hesterberg and coworkers suggest to differentiate “New Technology Diesel Exhaust” (NTDE, defined as DEE) from post-2006 and traditional diesel exhaust (TDE) (Hesterberg et al., 2011, 2012; McClellan et al., 2012). Although, DEE exposures in developed countries changed strongly during recent years, reliable animal experiments or epidemio-logical studies concerning the use of new fuels and technologies are almost lacking.

In order to assess the current situation, methods are needed which yield information cost effectively and in a fairly short time. This can be done by direct measurement of the exhaust components. However, it has been shown that the available analytical methods do not necessarily depict the complete picture of health hazards that are associated with DEE (Bünger et al., 2007; Krahl et al., 2008). Several investigators additionally studied biological *in vitro* effects which are believed to display DEE-associated health hazards. Therefore, data from chemical analyses and bio assays build the main part of this review supplemented by experimental studies in laboratory animals as well as one epidemiological cross sectional study in biodiesel exposed humans to give a preliminary answer to the question how biodiesel alters DEE.

## Methods

The search strategy included the following conditions: Original manuscripts published prior to 30 April 2011 were included when dealing with DEE from combustion of common DF and biodiesel, blends of biodiesel with DF (such as B20), or other biogenic diesel fuels. The search was applied to the following databases:

Pubmed (http://www.ncbi.nlm.nih.gov/sites/entrez), Toxline (http://toxnet.nlm.nih.gov), Environmental Science Network (http://www.osti.gov/esn), Web of Science (http://apps.isiknowledge.com), and Society of Automotive Engineers (SAE) (http://www.sae.org/jsp/jsps/advancesearch.jspSAE).

Emission studies from combustion of plant- and animal-derived biogenic diesel fuels were identified using the key words fatty acid methyl esters (FAME), vegetable oil (VO), hydrotreated vegetable oil (HVO) and the specific kinds of fuels, e.g. rapeseed methyl ester (RME), soy methyl ester (SME). These key words were combined with combinations of the search terms “health/toxicity/mutagenicity/and engine/exhaust/emissions”.

The studies which were evaluated concerning the regulated emissions (HC, CO, NO_*x*_, and PM) met the following criteria: emissions should have been measured during a certified test cycle and compared to DF as reference. However, studies not meeting the criteria for regulated emissions were considered if they comprised measurements of non-regulated emissions (aldehydes, ketones, aromatic compounds, PAH, nPAH), investigations in humans, data of animal experiments, or *in vitro* assays of biological effects. *In vitro* studies were only included if the assays directly compared biofuels to fossil diesel fuels and all tested fuels were combusted under identical conditions (test engine, test cycle, after-treatment). All the results for the biofuels were compared exclusively to the results of DF from the same study. This was necessary since assay protocols, methods used for the generation of the exhaust, as well as the sampling and extraction of the exhaust components varied enormously prohibiting a summarizing evaluation.

## Results

### Chemical analyses of regulated emissions

**Figure 2:**
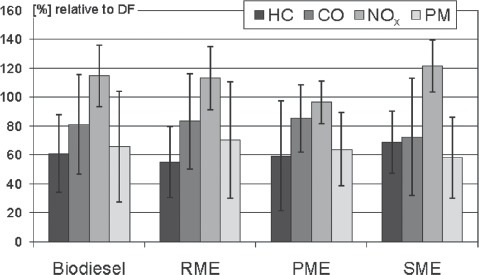
Comparison of the regulated exhaust constituents of biodiesel fuels (B100) in total and splitted by vegetable oil sources rapeseed methyl ester, RME), palm methyl ester (PME), and soybean methyl ester (SME) relative to DF (= 100%). Summarized are means and standard deviations from 104 engine test runs. Since biodiesel data mainly comprises RME emissions both charts are very similar. References: Aakko et al. (2000); Bouche et al. (2000); Fontaras et al. (2009, 2010a); Graboski et al. (1996); Hasegawa et al. (2007); Kawano et al. (2008); Kegl (2008); Knothe et al. (2006); Kousoulidou et al. (2009); Krahl et al. (2005, 2007a,b,c, 2008); May et al. (1997); McCormick et al. (2005); Munack et al. (2003, 2007); Nigro et al. (2007); Peterson et al. (2000); Ruschel et al. (2005); Schäfer (1996); Schumacher et al. (1996); Sharp (1996, 2000, 2005); Wirawan et al. (2008).

There are numerous analytical data regarding regulated DEE from the combustion of biodiesel. Among the exhaust components which are legally regulated in the European Union and the United States of America DEE from Euro II up to Euro IV engines show a reduction of emissions for HC, CO, and PM compared to DF. However, nitrogen oxides (NO_*x*_) are consistently increased (Figure 2). The results show a high variability which is mainly attributable to the differing engines and test cycles.

The kind of VO which is used as raw material for the biodiesel production has only a small impact on the regulated emissions. In some studies using older engines RME combustion yields even higher PM emissions compared to DF (Bünger et al., 1998; Bünger et al., 2000b). This observation is attributable to a high amount of unburned RME in the emissions of these engines, since differentiation between soluble and insoluble particle mass, reveals that only the soluble fraction is enhanced, whereas the solid fraction (soot) remains unaffected (Bünger et al., 2006; Krahl et al., 2007b).

**Figure 3:**
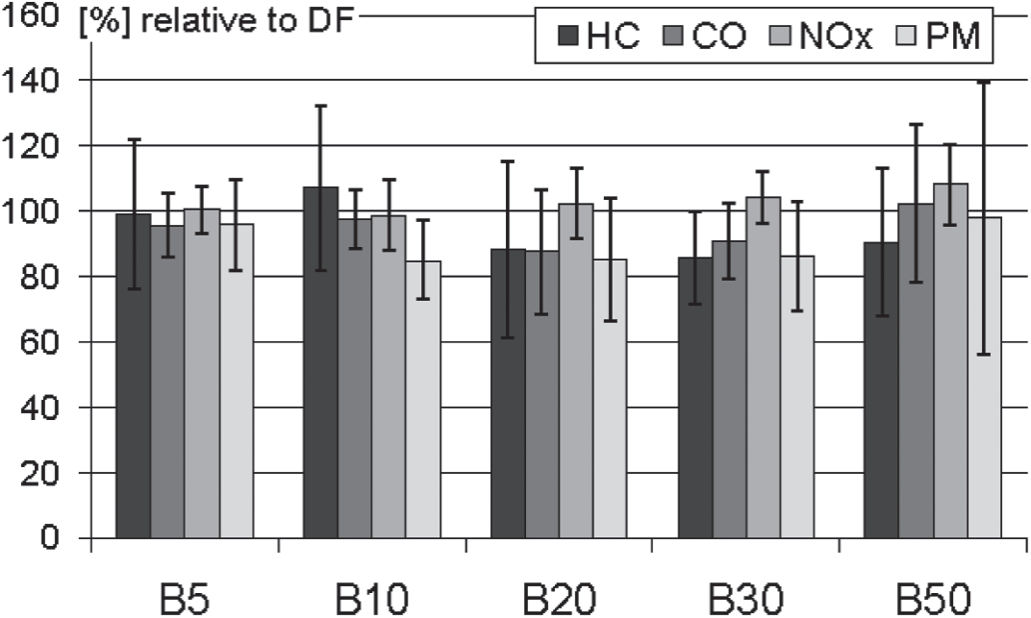
Regulated emissions of various biodiesel blends vs. DF (= 100%). Summarized are means and standard deviations of 245 engine test runs. References: Aakko et al. (2000); Alam et al. (2004); Arapaki et al. (2007); Clark et al. (1999, 2010); Frank et al. (2004); Hasegawa et al. (2007); Karavalakis et al. (2007, 2010a); Kawano et al. (2008); Kim and Choi (2010); Krahl et al. (2008); Lance and Andersson (2003); Luján et al. (2006); McCormick et al. (2005); Moser et al. (2009); Munack et al. (2007); Nigro et al. (2007); Peterson et al. (2000); Schäfer (1996); Sharp et al. (2000); Sze et al. (2007); Tompson et al. (2004); Turrio-Baldassarri et al. (2004); Wang et al. (2000); Wirawan et al. (2008); Yoshida et al. (2008).

European and US authorities aim on a broader market share of biofuels and try to achieve this intention by propagation of the use of biodiesel blends. The blending of fuels can change their physical and chemical properties and might as well change their characteristics of combustion. By now, also numerous data are available regarding the emissions of biodiesel blends. The most frequently investigated blends contain 5 and 20% biodiesel (B5 and B20). B5 shows only small effects on the regulated emissions compared to pure DF. A consistent reduction of HC is observed for all other blends. CO and PM are diminished in B10, B20, and B30; but B50 shows no differences for both emission types compared to DF. This effect may be caused by the small data basis for B50 indicated by the high standard deviation for PM. Blends from B20 up to B50 show an increase of NO_*x*_ becoming most apparent for B50 (Figure 3). Of all regulated emissions of the blends only the reduction for PM is significant, since standard deviations do not include the line marking 100% of DF emissions. Thus, trends are observed but specific engines may produce markedly differing emissions when fuelled with blends.

### Chemical analyses of non-regulated emissions

**Figure 4:**
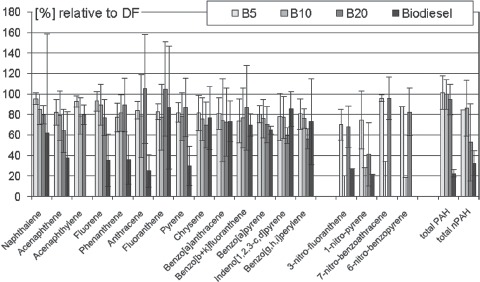
PAH- and nPAH-emissions of DEE from pure biodiesel and frequently investigated biodiesel blends compared to DF (= 100%). Summarized are means and standard deviations of 82 engine test runs. References: Karavalakis et al. (2009b, 2010b, 2011); Kooter et al. 2011; Munack et al. (2010, 2011); Ratclif et al. (2010); Song et al. (2011); Tang et al. (2007).

Non-regulated emissions of PAH and nPAH from combustion of biodiesel are generally below those of pure DF (Figure 4). Significant decreases are observed for most PAH from pure biodiesel. However, no consistent pattern can be observed regarding the blends, but there is a non-linear trend to lower emissions with increasing biofuel content for most PAH. B5 (5% biodiesel) leads to the most significant PAH emissions with the exception of 2-nitroanthracene and 6-nitrobenzopyrene; whereas B20 exhaust shows the lowest emissions with the exception of acenaphthene, fuorene, indeno[1,2,3-c,d]pyrene and 1-nitropyrene. Lower PAH and nPAH content should result in a comparable lower bacterial mutagenicity of biodiesel exhaust. However, this consistency does not apply to the blends, in particular not to B20 which shows an increased mutagenic effect (data shown in the mutagenicity section). No clear differences were observed regarding the various plant oil sources of the fuels, but data basis was too small to show minor changes.

**Figure 5:**
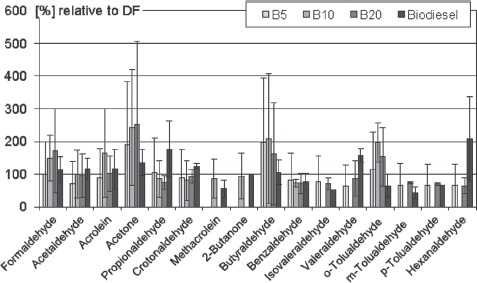
Emissions of aldehydes and ketones from pure biodiesel and biodiesel blends in relation to DF (= 100%). Summarized are means and standard deviations of 67 engine test runs. References: Fontaras et al. (2009, 2010b), He et al. (2009); Karavalakis et al. (2009a,b); Munack et al. (2010, 2011); Peng et al. (2008); Ratclif et al. (2010); Yuan et al. (2009).

The content of formaldehyde, acrolein, acetone, butyraldehyde, and *o*-tolualdehyde in the exhaust of biodiesel blends exceeds the content in DF up to 2.5-fold, but the standard deviations indicate a high variability of most results (Figure 5). Up to two-fold elevated emission from combustion of pure biodiesel is observed for acetone, propionaldehyde, crotonaldehyde, valeraldehyde, *o*-tolualdehyde, and hexanaldehyde. Concentrations of methacrolein, 2-butanone, benzaldehyde, isovaler-aldehyde, *m*-tolualdehyde *p*-tolualdehyde are slightly lowered in DEE from biodiesel and the blends compared to DF. Comparison of results for the pure biodiesel and the blends shows no consistent pattern, but interesting reverse trends are observed. In some substances with increased levels for the blend, the pure fuel shows no or only slightly enhanced levels, e.g. formaldehyde. On the other hand, aldehyde emissions showing a strong increase with pure biofuel (propionaldehyde, valeraldehyde, hexanaldehyde) reveal no elevated results for the blends. These contradictory trends are hard to interpret and may be caused by chance in the light of a relatively small data basis. In recent studies, markedly lower emissions are reported compared to earlier investigations. New engine technology has probably contributed to these results as well as the use of improved fuels.

### Effects in humans

A cross sectional study involved 763 male employees in road maintenance services which were exposed towards RME (*n* = 381) and DF exhaust (*n* = 382) (Hasford et al., 1997). A questionnaire was conducted concerning symptoms of the upper airways (cough with and without expectoration, feeling tightness of the chest, breathless-ness), mucous membrane irritation of the eyes, nose, and throat, a history of cardiovascular diseases as well as airway sensitization. Lung function was investigated in a small part of the employees. The exposure was estimated from the job titles and graded as weak, intermediate and strong; no exposure measurements were performed. Smoking habits, alcohol consumption, social status and dislike of the odor of the exhaust were evaluated as confounders.

The following symptoms were reported, regardless whether the study participants were exposed to exhausts from RME or DF: burning and watering eyes, rhinitis, cough, breathing difficulties, headache and nausea. Significant exposure-related health complaints were observed for respiratory symptoms [Odds Ratio (OR) = 2.2, 95% confidence interval (95% CI) = 1.3–3.8], irritation of mucous membranes (OR = 2.2, 95% CI = 1.3–3.7), impaired general condition (OR = 1.9, 95% CI = 1.1–3.4), subjective impairment by the exhaust (OR = 3.4, 95% CI=1.7–6.8), and aversion to the odor (OR = 2.0, 95% CI = 1.2–3.5). Odds ratios were calculated comparing individuals exposed to RME versus DF-exposed subjects.

When effects of RME emissions were compared with DF only a subjective impairment by the exhaust (OR = 2.4, 95% CI = 1.3–4.5), and aversion to the odor (OR = 2.0, 95% CI = 1.3–3.2) were significantly associated with RME exposure, whereas impaired general condition was reported significantly less frequently (OR = 0.6, 95% CI = 0.4–0.9). In case of “aversion to the odor”, the study participants also reported significantly more often subjective impairment. The authors concluded that most complaints were secondary effects due to the subjective aversion to the odor.

In an embedded cross-shift study, 46 individuals were investigated by lung function testing prior to and after exposures to DF or RME (Hasford et al., 1997). Participants were between 22- and 61-year-old (mean age: 42 years), 19 (41%) were smokers, 7 (35%) ex-smoker and 11 (24%) never smoked. Tree study participants reported diagnoses of bronchial asthma, two individuals reported chronic bronchitis and nine airway sensitization. No significant differences between lung function parameters were seen after exhaust exposures from DF or RME.

This study showed weak differences between effects of DF and RME exhaust. These were most probably linked to the odor of the biodiesel exhaust which was more often perceived as displeasing. Today, this problem is overcome by the use of diesel oxidation catalysts (DOC) minimizing the unpleasant odor of biodiesel exhaust. However, this study was not able to show chronic or sub-chronic effects of DEE due to the cross sectional design. Thus, comprehensive cohort studies should be started addressing outcomes like cardiovascular and respiratory diseases including lung cancer from combustion of biofuels. These studies should also result in data on the effects of new technology diesel engines as mentioned by Hesterberg and coworkers.

We included this study although it has the described limitations and was not published in a peer reviewed journal since it is the only epidemiological study directly comparing effects of biodiesel and DF and human data are believed to have particular significance.

### Risk assessment in humans

Health risks from DEE were assessed for inhabitants of the “South Coast Air Basin” in southern California (Morris et al., 2003). The alteration of health risk by use of B20 was calculated based on “Unit Risk Factors” of the following six exhaust components: benzene, 1,3-butadiene, acetaldehyde, formaldehyde, standard diesel particles and B20 diesel particles. According to the authors these compounds contribute to 90% of the health risk, which is caused by air pollution in this region and heavy-duty vehicles ought to contribute with 53% to the entire PM emissions of all trucks in this region. B20 would therefore reduce PM emissions by about 5%. According to a calculation of Lindhjem and Pollack (2000), the use of B20 should lead to a reduction of emitted particle mass by 9% and consequently to a reduction of PM toxicity by 5%. Based on these assumptions, Morris et al. (2003) estimated that B20 would lead to 2% less deaths if 50% and to 5% less deaths when all of the heavy-duty vehicles were run on B20.

The authors of this assessment made several assumptions which cumulate to a considerable uncertainty. Besides the notion that the procedure used in this study is not uniformly accepted and the unit risk factors are already estimates, it is not known if these six compounds really contribute to 90% of the health risk which is associated to air pollution in this region. For example, the contribution of PAH and nPAH to overall health risk was ignored. According to EPA (2002a), PAHs and their derivatives comprise <1% of the DPM mass, but many of these hydrocarbons are known to have mutagenic and carcinogenic properties according to evaluations by the International Agency for Research on Cancer (IARC). In addition, unexpected effects occur when biodiesel in certain amounts is added to common DF (blending). Krahl et al. (2008) observed an increase of the mutagenic potency when combusting blends showing the maximum using B20. This effect occurred when biodiesel was mixed with different petroleum-derived fuels and was reproduced using three separate engines.

### Animal experiments

Animal experiments directly comparing the effects of DF and biodiesel exhaust were not identified, but we found subchronic studies which investigated DF and biodiesel using comparable conditions as well as similar toxi-cological and histopathological endpoints at Lovelace Respiratory Research Institute, Albuquerque, NM, USA (Table 1) (Finch et al., 2002; Reed et al., 2004, 2005, 2006). SME exhaust was investigated in male and female F344-rats. The rats were exposed by inhalation for 13 weeks, 6 h/day and 5 day/week to dilutions of SME emissions. Pure SME was combusted in two turbocharged six cylinder, 5.9 L Cummins ISB diesel engines (1998) which were used alternately during the study (Finch et al., 2002). The engines were operated according to the 20-min US EPA Heavy-Duty Engine Dynamometer Schedule (40 US CFR, Part 86). The engine exhaust was led through a dilution tunnel and diluted ∼10-fold. A portion of the diluted BDE was extracted from the tunnel and diluted serially to the exposure concentrations. The administered dose range was orientated on toxicity of NO_*x*_ and dilutions were made to yield 5, 25, and 50 ppm NO_*x*_. The NO_*x*_ concentrations were similar to those reported for high concentrations of contemporaneous mineral diesel exhaust. These levels corresponded to particle masses (PM) of 0.04, 0.2, and 0.5 mg/m^3^. According to the authors, this exceeds the particle mass which is found in ambient air by about two orders of magnitudes and those which can be found at “worst-case” conditions at workplaces by about one order of magnitude.

**Table 1:** Overview of toxicological and histopathological findings in subchronic inhalation studies using F344 rats.

Results	Exposure			
	Soya Methyl Ester (SME): 40, 200, 500 μg/m^3^ PM Engine model year: 1998 (Finch et al. 2002)	Mineral diesel fuel: 30, 100, 300, 1000 μg/m^3^ PM Engine model year: 2000 (Reed et al. 2004)	Mineral diesel/water emulsion: 100, 200, 400 μg/m^3^ PM Engine model year: 2001 (Reed et al. 2005)	Mineral diesel/methanol–water emulsion: 125, 250, 500 μg/m^3^ PM Engine model year: 2002 (Reed et al. 2006)
Clinical chemistry	Blood urea nitrogen ↓ Alkaline phosphatase ↓ Lymphocytes and monocytes in peripheral blood ↓	Serum cholesterol, treatment related ↓ (at 6 month n. s.) γ-GT ↑. Blood urea nitrogen ↑ White blood cells ↓	Serum cholesterol ↓ ♂ high level, ♀ mid and high level γ-GT ↑ ♂ high level recovery group Blood urea nitrogen ↓♂ mid and high level, high level recovery White blood cells ↑ ♂ low and ♀ mid level and high level recovery group	Serum cholesterol ↓ high level** γ-GT ↑ ♀ mid and high level recovery group Blood urea nitrogen ↑ ♂ mid & high level Alkaline phosphatase ↓ ♂ high level, ↑ ♀ mid level White blood cells, Lymphocytes ↑ ♂ mid and ♀ low level
Pathology	Lung weight ↑ high level, lung/bw ratio ↑, high-level ♂ (♀ ↑ n. s.) Discolored lungs ↑, treatment related Liver weight of high-level rats ↓	Lung weight ↑ ♂ Lung volume ↑ (♂ n. s.) Kidney weight ↑ Lung/bw ↑ ♀ high level Discolored lungs ↑ Liver weight ↓ ♀ high, ♂ mid and high level kidney/bw ↑ ♂ high level	Discolored lungs, high level	
Histology	Treatment related elevation of alveolar macrophages* and PM in macrophages Single rats: alveolar hyperplasia and histiocytosis, minimal bronchiolar metaplasia ♂ high level	Treatment related elevation of alveolar macrophages* and PM in macrophages	Treatment related elevation PM in alveolar macrophagesMinimal alveolar macrophage hyperplasia ↑	Treatment related elevation PM in alveolar macrophagesMinimal alveolar macrophage hyperplasia

Only data are shown which are significant unless otherwise stated (n. s.) and consistent in regard to the level of exposure. Significant findings which only occurred in single studies are not shown, with the exception of the histological data. Results occurred in males and females, unless otherwise stated. γ-GT: gamma-glutamyl transpeptidase; bw: body weight.

* Neutrophils were not associated with the elevated alveolar macrophages.

** A “No Observed Adverse Effect Level” (NOAEL) was deduced from serum cholesterol levels which were unaffected at 250 μg/m^3^.

Exposures were generated using engines from one series of the same manufacturer (5.9 L Cummins ISB turbo diesel). The varying model years are indicated in Table 1. The studies investigated exhaust of contemporary US certification petroleum diesel fuel (Reed et al., 2004), a mineral diesel/water emulsion (Reed et al., 2005) and exhaust of a mineral diesel/methanol–water emulsion (Reed et al., 2006). Concentrations of each study difered; exact exposure concentrations are displayed in Table 1. Reed et al (2004) applied a “variable-load heavy-duty test cycle” and Reed et al. (2005 and 2006) a “continuous, repeating, heavy-duty certification cycle” (both according to US Code of Federal Regulations, Title 40, Chapter I). In the first study, F344-rats were exposed to DEE for 6 hours per day, 7 days per week, either for 1 week or 6 months. The second study investigated emissions from the combustion of PuriNO(x)™ diesel fuel/water emulsion which were diluted with air to exposure concentrations of 100, 200, and 400 μg/m^3^ PM. F344-rats were exposed to exhaust atmospheres for 6-h a day, 5 days/week for the first 11 weeks and 7 days per week thereafter. Exposures ranged from 58 to 70 days, depending on the treatment group (Reed et al., 2005). Reed et al. (2006) exposed F344 rats towards 125, 250 and 500 µg/m^3^ PM. 6-h a day, 5 days/week for the first 11 weeks and 7 days/week thereafter. Exposures ranged from 61 to 73 days depending on the treatment group. Treatment duration varied in the latter studies (Reed et al., 2005, 2006), because investigations of reproduction and development were included.

The study design for the described inhalation studies included a large number of identical read out parameters: Fifteen rats of each sex were used for general histology, hematology and clinical chemistry, gross necropsy and histopathology. Five male and female rats were investigated concerning “glial fibrillary acidic protein” (GFAP), micronuclei (MN) and sister chromatid exchange (SCE). Analysis of MN and SCE was only done in the high exposure groups.

Table 1 displays notable results of clinical chemistry, pathology and histology in detail. Exposure-related clinical effects consisted in a significant and concentration-related reduction of serum cholesterol and elevation of γ-glutamyltranspeptidase (γ-GT) in DF exposed rats (Reed et al., 2004, 2005, 2006). This was not seen in the study which investigated SME exhaust (Finch et al., 2002). Till date a clinical relevance of these observations for humans is not known, but the reproducible elevation of γ-GT suggests a relevant toxic effect on the liver tissue. Exposure-related pathological results were restricted to the lungs of the highly dosed rats and were most pronounced among females. This is consistent with the notion that female rats are more sensitive towards PM than males (ILSI Risk Science Institute Workshop Participants, 2000). The mean lung/body weight ratio of the high-level females was greater in the exhaust treated groups (Finch et al., 2002; Reed et al., 2004, 2005). This was partly in-agreement with the concentration-related increase of gross necropsy findings in discolored lungs (except Reed et al., 2005) and elevated numbers of alveolar macrophages and particle inclosing alveolar macrophages which was seen in all of these studies. These particles were interpreted as diesel engine particles. Minimal bronchiolar metaplasia in the alveolar ducts was seen in three high dosed females exposed to SME exhaust (Finch et al., 2002). This was interpreted as response to epithelial injury. Pursuant to the recovery groups, these findings seem to be reversible. No treatment-related effects on MN and SCE rates were seen.

The non neutrophilic inflammatory effect caused by SME exhaust (Finch et al., 2002) seems to be stronger than the effect of DF (Reed et al., 2004, 2005, 2006), although the reviewed subchronic inhalation studies are not directly comparable. However, this stronger effect is probably caused by exhaust constituents that are specific for biodiesel because the generation of exposures was very similar using SME and DF. The generally higher emissions of aldehydes and ketones of biodiesel (Figure 5) may explain this stronger irritant effect of SME exhaust. Decreasing serum cholesterol is the serum parameter most consistently associated with exposure to DF exhaust. But it is unclear if this finding is of patho-physiological relevance for human health, since a disease due to low cholesterol is not known. Thus, the lack of this observation for SME exhaust cannot be interpreted as an advantage for this fuel.

In conclusion, the study of Finch and colleagues (2002) demonstrated that 100% soybean-derived fuel showed only modest inflammatory effects at the highest exposure level. However, this is as well true for the fossil diesel fuels which were investigated in the studies of Reed et al. (2004, 2005, 2006). Direct comparison of biodiesel and DF in the same study protocol is necessary to clarify if biodiesel induces less infammation of the lung.

A recent study was published directly comparing effects of inhalation exposure to DF, B50, B100 from soybean ethyl esters (SEE) in Balb/c mice (Brito et al., 2010). The animals were exposed to emissions from a diesel electrical generator (BD 2500, CFE, Branco) at constant load for 1 h. All exposures were adjusted to PM_2.5_, 550 μg/ m^3^. Read out parameters were collected from pulmonary and cardiovascular system, blood, bronchoalveolar lavage (BAL), and bone marrow. Compared to DF, B100 caused a significantly increased heart rate variability, but no changes in heart rate or blood pressure. MCV and platelets were elevated possibly indicating an enhanced thrombogenic potency of the B100 exhaust. All other blood parameters remained unchanged or gave inconclusive results. Pulmonary and systemic infammation was increased by all fuels without significant differences. Thus, the conclusion of the authors that biodiesel has stronger effects on pulmonary and systemic infammation is not supported by the data. In addition, the publication has a misleading title. Mice were not only exposed to “diesel and biodiesel particles” but also to the gaseous phase of the emissions (Brito et al., 2010).

### *In vitro* studies investigating mutagenicity and DNA damage

All investigators except Morin et al. (2000) used filter sampled diesel exhaust particles (DEP) for the *in vitro* assays which were extracted with solvents (Table 2). The particle mass (PM) is usually determined by weighing the conditioned filters before and after sampling. The extracted organic matter is determined by calculation of the difference between the filter weight prior to and after the extraction. Most *in vitro* studies investigated bacterial mutagenicity as a paradigm of the carcinogenic potential of DEP. Mutagenic effects are attributed to their content of PAH and nPAH. Bacterial mutagenicity of diesel exhaust particle extracts was already shown by Huisingh et al. (1978) and thereafter by many other researchers. Most studies comparing exhaust of DF and FAME used the bacterial reverse mutation assay (so called Ames test; Ames et al., 1973, 1975). This assay is particularly suited as screening tool for hydrophobic mutagenic compounds or mixtures such as PAH from DEE. It is used worldwide and adopted by the OECD as guideline 471. In contrast to mammalian cells, the bacteria tolerate high amounts of the solvent dimethyl sulfoxide (DMSO). This is a particular advantage for the investigation of water insoluble compounds such as PAH and nPAH. The good reproducibility of this test reduces the probability of results which are caused by chance. Moreover, a great amount of tests can be performed to comparably low costs and false positive results are rare. Bacterial mutagenicity of DEE is differently pronounced in *Salmonella typhimurium* tester strains. The tester strains TA98 (frame-shift mutation) and TA100 (base-pair substitution) are particular sensitive (Claxton, 1983).

**Table 2:** *In vitro* studies of biological effects from DEE after combustion of biodiesel (B100) and biodiesel blends compared to DF.

Authors	Cells/Tissues	Endpoints	Engine	Fuels	Test cycles	Extraction	Biological effects	Remarks
Grägg 1994	*S. typhimurium* (TA98, TA100) *Sub-cellular*	Bacterial mutagenicity Ah- receptoraffinity	11 L Scania DSC 1127 truck diesel engine	DF, DF with 2000 ppm ethyl-hexylnitrate, MK1, MK2, RME, B5, B30	City-Line Cycle, Univ. Brunsvig, Germany, ECE R 49	No description of details	RME particle extracts up to sixfold less mutagenic than DF.Blends produced stronger mutagenicity than pure fuels.No coherent results from the Ah-receptor assays.	Results not sufficiently documented. Ah-receptor-assay of questionable value concerning DEE
Carraro et al. 1997	*S. typhimurium* (TA98, TA100)	Bacterial mutagenicity	1.9L DI and IDI turbo diesel, EGR	DF with 1000 ppm sulfur, RME	European standard test cycle (ECE-EUDC)	Soxhlet with benzene and acetone	RME up to two-fold less mutagenic than DF, good correlation with PAH and nPAH analyses.	Methods and results of chemical analysis not sufficiently documented.
Bagley et al. 1998	*S. typhimurium* (TA98, TA98NR, TA98/1.8–DNP6)	Bacterial mutagenicity	1983 7L Caterpillar 3304 PCNA Diesel engine± DOC	DF, SME	light- and heavyduty transient test cycles of the U.S. Bureau of Mines (USBM)	Soxhlet with DCM, 24h in the dark	Mutagenicity of particle extracts stronger compared to condensates. SME with DOC up to four-fold less mutagenic than DF. DOC reduced mutagenicity of SME and DF to a comparable extent.	PM and PAH with SME reduced, SOF slightly increased.
Bünger et al. 1998	*S. typhimurium* (TA97a, TA98, TA100, TA102) Mice fibroblasts (L929)	Bacterial mutagenicity Cytotoxicity	1.9 L DI VW turbo diesel engine, EGR, DOC	DF, RME	US- FTP-75. European MVEG-A, incl. cold start (MVEG-A1).	Soxhlet with DCM, 24h in the dark	RME up to five-fold less mutagenic than DF, strongest mutagenicity under cold start conditions. RME stronger cytotoxic than DF.	RME led to an increase of soluble PM.
Bünger et al. 2000a	*S. typhimurium* (TA98, TA100)	Bacterial mutagenicity	One Cylinder test engine Fary-mann 18D	DF, LS-DF, RME, SME	ECE R 49	Soxhlet with DCM, 12h in the dark	Mutagenicity of DF about two-fold stronger compared to SME, LSDF, and RME. Sulfur content and high engine load were associated with strong mutagenicity of PM.	PAH content in DF- and SMEexhaust increased compared to LSDF and RME.
Bünger et al. 2000b	*S. typhimurium* (TA98, TA100) Mice Fibroblasts (L929)	Bacterial mutagenicity Cytotoxicity	4 Cylinder Fendt 306 LSA tractor-engine	DF, RME	ECE R 49	Soxhlet with DCM, 12 h in the dark	DF at heavy load about four-fold stronger mutagenic. RME at partial load about four-fold stronger cytotoxic.	RME: TPM↑ SOF↓ DF, at partial load: trend towards ultrafine particles ↑
Morin et al. 2000	Rat lung slices	Apoptosis and biochemical markers of inflammation	1 Cylinder diesel test engine, 230 ccm	DF, RME, and B30	Engine operation at 3000 min^-1^	No extraction, direct exposure to 5, 10, 15, 25, 60, and 85% filtered and unfiltered exhaust for 3 hours	No clear trend for TNFα.GSHdepletion: up to 70% with un-filtered and 30% with filtered exhaust. DF led to apoptosis, RME and the blend did not.	Inconclusive results. The impact of apoptosis concerning diesel exhaust is not clear; it may be beneficial in case of genotoxic effects.
Kado et al. 2001	*S. typhimurium* (TA98)	Bacterial mutagenicity	5.9 L Cummins ISB turbo diesel, ± DOC	DF, B20, B50, B100 from Rapeseed ethyl ester (REE),	US Code of Fed Reg. 40, Part 86, cold- and hot-start	Sonication with dichloromethane (DCM)	B100 (REE) much less mutagenic than DF. B20 had strongest mutagenicity. B20 and B50 with DOC up to 10-fold stronger mutagenic than without DOC.	Results for increased bacterial mutagenicity with DOC for B20 and B50 is not discussed in the paper
Kado and Kuzmicky 2003	*S. typhimurium (TA98)*	Bacterial mutagenicity	6 Cylinder. DI 11.1 L Detroit turbo-diesel	DF, CME, SME, PLME, YGME, and BTME	US Code of Federal Regulations 40. Part 86. Subpart N	DCM (no details given)	PM and mutagenicity at cold- stronger compared to hot start. Reverse mutations /KWh: DF > CME > SME = PLME = YGME > BTME.	Biofuels from animal fat showed strong direct mutagenicity.
Bünger et al. 2004	*S. typhimurium* (TA98, TA100)	Bacterial mutagenicity	VW 1.9 L-TDI, ± DPF and during regeneration	DF, RME		Soxhlet with DCM, 12h in the dark	With DPF: mutagenicity↓ Regeneration: mutagenicity↑, up to 2.5-fold stronger with the aged catalyst, most pronounced for DF.	PM was reduced by DPF and increased during regeneration phase of the DPF.
Turrio-Baldassarri et al. 2004	*S. typhimurium* (TA98, TA100)	Bacterial mutagenicity	IVECO 8360. 46R, 7.8 L turbodiesel, Euro II	DF, B20	ECE R 49	ASEextractorfilter: toluene, PUF: 1/1*n*-hexane/acetone	Pure fuels and blends did not differ concerning mutagenicity.	Strong variability of the Ames-Test results. Extraction at high temperature and pressure.
Bünger et al. 2006	*S. typhimurium* (TA98, TA100)	Bacterial mutagenicity	One Cylinder test engine Fary-mann K54, ± DOC	DF, LSDF, RME, SME	ECE R 49	Soxhlet with DCM, 12h in the dark	With DOC mutagenicity up to 20%↓ At heavy load about 70% ↑ in case of RME and SME, at idling heterogeneous results.	Stronger mutagenicity with DOC under certain conditions; possibly correlated with NO_x_.
Krahl et al. 2006	*S. typhimurium* (TA98, TA100)	Bacterial mutagenicity	5.9 L IVECO Turbodiesel tector F4A with SCR-System	DF, RME, RME with content of 10 ppm phosphor (RME_10_)	E46 Endurance test and European standard cycle (ESC)	Soxhlet with DCM, 12h in the dark	No significant mutagenicity was detected with the brandnew SCR. Slight mutagenic effects were observed after an engine operation of 1000 hours. No influence of phosphor content on mutagenicity.	SCR seems to be a very effective exhaust after-treatment. Good durability of the SCR towards phosphor.
Ackland et al. 2007	Human alveolar cells (A549)	Apoptosis	1.6L VW-engine	DF, RME, and blends thereof	ECE Euro 2	Three days in DCM (no further details)	Induction of apoptosis was stronger for DF than for RME, strongest effect observed for B20.	The impact of apoptosis concerning adverse health effects of DEE is unclear.
Bünger et al. 2007	*S. typhimurium* (TA98, TA100)	Bacterial mutagenicity	Mercedes OM 906 LA 6.4 L turbo-diesel, Euro III	DF, RME, Rapeseed vegetable oil (RVO)	Stationary European cycle (ESC)	Soxhlet with DCM, 12 h in the dark	Weak mutagenicity of PM extracts and condensates from DF and RME, 9.7– up to 59-fold stronger mutagenicity in TA98, 5.4– up to 22.3-fold in TA100, condensates of the RVO fuels caused an up to factor 13.5 stronger mutagenicity	RVO was combusted with and without preheating to 70°C (two-tanktechnology). The engine itself was not adapted for RVO combustion.
Krahl et al. 2008	*S. typhimurium* (TA98, TA100)	Bacterial mutagenicity	Mercedes OM 906 LAMAN D08 36 LFL51 turbo-diesel, 6.8 L, DPF, Euro IVAVL502.019, 1.5 L	DF, B5, B10, B20, B30, B40, B50, B100 from RME	ESCEuropean Transient Cycle (ETC)Rated power	Soxhlet with DCM, 12 h in the dark	Blends showed increased mutagenicity with three enginesStrongest effect was observed for B20 (up to three-fold compared to the pure fuels): B20 > B10 > B50 = B5 > DF > RME.	
Liu et al. 2008	*Vibrio fischeri*, BEAS-2B cells (human bron-chial cell-line)	Microtox-test MTT- assay	Dieselgenerator, 13 kW	DF, B10, B30, B50, B75, B100 from PME	constant load	Soxhlet with DCM and *n*-hexane	PME yielded increased toxicity, most pronounced for B50.	PME caused increased PM emissions.
Jalava et al. 2010	Mouse RAW 264.7 macrophages	COMET Assay, apoptosis, cytotoxicity, ROS, TNF-α	1.1 L IDI Kubota D1105-T Diesel, EURO II	DF, RME, HVO	ISO standard steady state cycle (8178–4:1996)	Sonication for 2 × 30 min. with methanol	Concentrationrelated DNA strand breaks and toxicity of the extracts, no differences regarding DF, RME, HVO, and use of DOC. DF and HVO stronger TNF-α induction, ROS induction by HVO and RME.	Detailed information about the used catalyst is lacking. All effects were referred to TPM and not to KW/h or exhaust volume.

In addition some studies investigated cytotoxicity, apoptosis, and Ah-receptor affinity of DEP. In one study, apoptosis and parameters of infammation were measured after direct exposure of rat lung slices to DEE. Table 2 summarizes the reviewed *in vitro* studies of biological effects after combustion of biodiesel (B100) and biodiesel blends compared to DF.

According to current guidelines, short time test require relatively high concentrations to compensate the short residence time of the test compounds compared to the *in vivo* situation. Unfortunately, a lot of different test protocols were applied making even studies hard to compare using the same endpoints. This started with strong differences in the generation of the exhaust (mainly concerning the engines and the test cycles), the use of exhaust after-treatment devices, the sampling and processing of the emissions and last but not least the different fuels (biofuels as well as DF qualities). Sulfur and aromatics content of DF ranged from 1 to 500 ppm. Sulfur and aromatic contents were lowered in recent years resulting in a strong reduction of PM and PAH emissions. The differences between the biodiesel qualities are mentioned in the manuscript.

Generally, the more recent studies revealed a clear reduction of genotoxic effects especially for DF showing less differences between the biofuels compared to reference fuels. In the earlier studies, these differences were more pronounced. This can mainly be interpreted as a result of the improvements of DF, such as lowering the sulfur content from more than 500 ppm in the 1980s to less than 10 ppm (Bünger et al., 2000a; Bünger et al., 2006; Hesterberg et al., 2006). Improvements of the diesel engines reduced toxic effects of emissions regardless of the combusted fuel, noted by Hesterberg and coworkers (2011): “New Technology Diesel Exhaust (NTDE) should be differentiated from traditional diesel exhaust (TDE)”. However, as a consequence of this tremendous technical and methodological variety, we only included publications which directly compared biofuels with DF within an identical study protocol.

First comparative investigations on DF, RME, and blends thereof were conducted by the Swedish Environmental Protection Agency in cooperation with the Karolinska Institute (Grägg, 1994). RME particle extracts were up to six-fold less mutagenic than DF in tester strains TA98 and TA100. Blends (B5 and B30) produced a stronger mutagenicity than could be expected from the results for the pure fuels. In all experiments, DEE-induced mutagenic effects which were stronger without addition of a metabolic activation system (S9-mix). Since bacterial mutagenicity of non substituted PAH requires metabolic activation, the positive results without metabolic activation confirm that components apart from non substituted PAH contribute significantly to mutagenic effects of particle extracts from DF and RME as well. This effect of so called direct acting mutagens in diesel exhaust was already observed in earlier studies and attributed to substituted PAH such as nPAH (Mermelstein et al., 1981, Hayakawa et al., 1997; Westerholm et al., 2001; Heeb et al., 2008). Nitrated-PAH can be formed by a reaction of PAH with the NO_*x*_ present in DEE. These substances show a strong direct mutagenicity, while their parent compounds are not or much less mutagenic (Ohe, 1984; HEI, 1995; Victorin, 1994).

The main results of the Swedish study by Grägg were confirmed in most succeeding studies investigating bacterial mutagenicity using the Ames test in engines up to the emission standard Euro IV. (Carraro et al., 1997; Bagley et al., 1998; Bünger et al., 1998; Kado et al., 2001; Bünger et al., 2006). Generally, mutagenicity of the exhaust was associated with PM and only to a minor extent with the gaseous or semi-volatile phase. Direct bacterial mutagenicity was stronger in most investigations compared to indirect mutagenicity. Using DF with low or very low sulfur content the mutagenicity of DEE was similar or even lower compared to RME (Bünger et al., 2007).

Carraro et al. (1997) observed less reverse mutations from combustion of RME, both with and without S9-mix. Reduced mutagenicity correlated with reduced amounts of total PAH. No details were given on the analytical methods and which PAH were measured. Bagley et al. (1998) found a stronger mutagenicity of particle extracts compared to condensates. Exhaust of SME was up to four-fold less mutagenic than DF. Exhaust after-treatment with a DOC reduced mutagenicity of SME and DF to a comparable extent.

Bünger et al. (1998) investigated bacterial mutagenicity of DEE from DF and RME using TA97a, TA98, TA100 and TA102. The particle extracts showed mutagenicity only with strains TA98 and TA100. DF exhibited an up to five-fold stronger mutagenicity compared to RME in TA98. These results were confirmed by the next study of Bünger et al. (2000b).

Also in 2000, Bünger et al. (2000a) published a study investigating the Influence of sulfur content of fuels on PM, PAH content, and mutagenicity of the resulting DEE after combustion. Two virtually sulfur-free biofuels (RME, SME) were compared to a “low sulfur” diesel fuel (LS-DF) containing 2 ppm sulfur and – at that time – common DF with 370 ppm sulfur content. At rated power mutagenic effects of particle extracts from all exhausts were observed in TA98, most pronounced for DF and 2- to 10-fold less for LS-DF, SME and RME. In summary RME, SME and LS-DF exhaust contained less soot, PAH and mutagenic compounds. In modern common diesel fuels the sulfur content is heavily reduced leading to PAH emissions and mutagenic effects at the same low level like biodiesel (Bünger et al., 2007).

Kado et al. (2001) investigated rapeseed ethyl ester (REE) for PAH emissions and mutagenic effects after combustion in a small truck running exclusively in Yellowstone National Park. Results from PAH emissions testing of the truck at 3,700 miles and 86,600 miles gave similar results. The B20 of REE had the highest mutagenic potency of all tested DEE (approximately double those measured for DF). The lowest mutagenic emissions were observed for pure REE regardless if tests were run with or without DOC. In general, the PM emissions measured for all fuels without DOC were approximately double than those measured with the catalyst. However, for both blends (B20, B50) the mutagenicity was up to 10-fold stronger when the engine was equipped with a DOC. The authors do not discuss these results, but similar findings were published by Bünger et al. (2006), showing an increase of mutagenicity for combustion of SME and RME when the engine was operated with a DOC under heavy engine load conditions. Bünger and cowork-ers hypothesized that the OCC increases formation of direct acting mutagens under certain conditions by the reaction of NO_*x*_ with PAH resulting in the formation of nitrated-PAH. Most of these compounds are powerful direct acting mutagens.

Kado and Kuzmicky (2003) compared DEE from various FAME of plant and animal origin and DF. Particle emissions were about three-fold higher for DF compared to the biodiesel fuels. Beef tallow methyl ester (BTME) caused the least SME the most particle emissions of the biodiesel fuels under cold start conditions. The ranking of PM emissions in descending order was as follows: DF > canola methyl ester (CME) > SME = pork lard methyl ester (PLME) > yellow grease methyl ester (YGME) > BTME. When investigated under hot-start conditions the ranking was as follows DF > SME > CME > BTME > PLME = YGME. Under cold start conditions the mutagenic effects for all bio fuels were smaller than for DF, smallest for SME producing a 3.5-fold lower mutagen-icity. Effects for PLME, BTME, and YGME were similar and two-fold lower compared to DF. CME produced the highest mutagenicity of the FAME but still 1.5 lower than DF. Mutagenicity under hot-start conditions showed no consistent pattern.

In three studies which included blends, stronger mutagenic effects of DEE from blends were reported compared to pure DF or biodiesel (Grägg 1994, Kado et al. 2001, Krahl et al. 2008). This effect was not seen in a study by Turrio-Baldassarri and coworkers (2004). In the study of Grägg (1994) blends of DF and RME caused stronger mutagenicity as would have been expected in regard to the portions of the basic fuels. DF with 5% RME induced about four-fold more reverse mutations compared to DF or pure RME. DF and 30% RME showed as well elevated mutagenicity but not consistent for all investigated conditions. In contrast to other investigations Kado et al. (2001) used REE and DF to produce B20 and B50 blends. B20 showed stronger mutagenicity than pure DF and pure REE when the engine was run with a DOC. Turrio-Baldassarri et al. (2004) compared mutagenic effects of the emissions of a city-line bus which was operated with DF or B20. RME served as the biofuel component. Since the results of two experiments strongly scattered, no clear trend was observed concerning the blend. At average exhaust of DF and the blend caused comparable effects. According to the authors variability of the bacterial reverse mutation test may account for these results.

The most detailed study on blends was conducted by Krahl et al. (2008). DF was blended with 5% up to 50% RME and combusted in three different engines. Combustion of three different DF qualities blended with 20% RME in a Mercedes OM 906 engine (Euro III) caused up to 70% stronger mutagenicity compared to the pure fuels. Combustion of B5 up to B40 in a MAN D08 36 engine (Euro IV) as well revealed up to two-fold increased mutagenicity of the particle extracts with a maximum at B20. When an AVL one cylinder test engine was fuelled with B5, B10, B20 and B50 blends, the blends again caused a up to three-fold higher mutagenicity and the strongest effect was induced by B20 again. However, it was shown that storage of blends of DF and biodiesel can lead to a formation of deposits. This effect was particularly observed in B20 blends (Fang and McCormick, 2006). Therefore, shorter storage times of the blends may explain the differing results of Turrio-Baldassarri and coworkers (2004). Also, antioxidants can prevent deposits of blends (McCormick and Westbrook, 2010).

Jalava et al. (2010) used the single cell electrophoresis (comet assay) to study the induction of DNA strand breaks by DF and RME in mouse RAW264.7 macrophages. Exhaust was generated according to the ISO standard steady state cycle (8178-4:1996) in a 1.1 L IDI Kubota D1105-T diesel engine which complied with EURO 2. However, samples were taken only at load modes up to 50% since the temperature in the dilution tunnel exceeded the ISO standard limits. The PM of the DEE was collected with a “high volume cascade impactor”. Polyurethane foam was used as collecting material, combined with a 0.2 μm PTFE bottom filter. Particles of the different particle sizes were pooled and suspensions were prepared by sonication in aqueous solution and 0.3% DMSO. The suspended particles (50, 150 and 300 μg) induced concentration-related DNA strand breaks and toxicity but no differences were observed between combustion of DF and RME. However, possible differences may have been masked because the authors calculated mutations per mg PM. Since combustion of DF led to higher PM emissions the number of mutations should have been corrected for the difference of PM between the fuels. Other authors avoided this problem by referring the effects to exhaust volume (L), driven distance (km) or provided energy (KW/h). The same holds true for the other parameters in this publication discussed below.

### *In vitro* studies investigating non mutagenic effects

Extracts of biodiesel exhaust were investigated concerning cytotoxicity in mice fibroblasts (L929) using the neutral red assay. In FTP-75 test cycle, RME particle extracts yielded stronger cytotoxicity compared with DF. The investigation of cytotoxic effects was limited by the amount of particle extracts which could be redissolved in DMSO, since mammalian cells do not tolerate growth media containing more than 2% DMSO (Bünger et al., 1998). The stronger cytotoxicity of RME exhaust was confirmed in a second study (Bünger et al., 2000b). There, RME caused four-fold stronger toxicity towards L929 cells at idling mode. The authors attributed the stronger toxicity of RME to a higher amount of carbonyl compounds in the exhaust.

Cytotoxic effects were also investigated by Liu et al. (2008). They used palm oil-methyl-ester (PME) and various blends (10, 30, 50, 75 and 100%). Toxicity *in vitro* was assessed using the MTT-test with human bronchial-epithelial BEAS-2B cells and the Microtox-test. PM, semivolatile compounds and toxicity of the particle extracts increased with increasing portion of biodiesel up to 50%. With larger portions of biodiesel PM and toxicity declined again. Pure DF yielded the least toxic exhaust. However, no clear trend was observed since toxicity was quite weak and did not exceed 80% vitality. These investigations add further evidence to the notion that blends can yield stronger toxic exhaust than the pure fuels.

Induction of apoptosis by RME, DF and blends (B20, B40, B60, and B80) was investigated in human alveolar A549 cells (Ackland et al., 2007). The particle extracts (25 μg/mL) induced multi nuclear cells: B20 caused 52% multi nuclear cells whereas only 16% were seen with the B80 blend (7% background). The weakest caspase 3 expression occurred in cells which were exposed towards the pure RME and B80. Based on caspase 3 expression and cleaved pan-cytoceratine, induction of apoptosis was more pronounced with pure DF compared to RME. Induction of ZnT3 was as well most pronounced by the B20 blend, amounting to approximately eight-fold above the background level. The authors concluded: “The increase in ZnT3 expression seen in apoptotic cells following DEE suggests a role for this zinc transporter in the apoptotic pathway, possibly through controlling zinc fuxes. As exposure to diesel exhaust particles is associated with asthma and apoptosis in airway cells, diesel exhaust particles may directly contribute to asthma by inducing epithelial cell death through apoptotic pathway.

However caspase 3 is as well involved in the activation of the proinflammatory cytokine IL-1β and ZnT3 might have been induced indirectly by glucose depletion in damaged cells. Conclusions concerning a direct apoptosis induced by DEE might be too far-reaching, since no cytotoxicity was measured and the extracts were applied to the cells in a medium containing 90% ethanol (ethanol concentrations at and above 1% in the medium are cytotoxic). However, concerning toxic effects of blends, this study revealed the same trend as previous studies applying different toxicological endpoints. The B20 blend tends to yield the strongest toxicity of all blends and stronger toxicity than the pure fuels.

Toxicity of DF-, RME- and B30-exhausts was investigated in rat lung slices (Morin et al., 2000). The lung slices were directly exposed with the filtered and diluted exhaust for 3 h. Intracellular ATP and GSH served as parameter of energy charge or cell vitality and reduction equivalents respectively, nucleosomes as parameter which depicts apoptosis, and extra-cellular TNFα (Tumor-necrosis-factor-α) to measure inflammatory reactions. ATP was only slightly reduced in the lung slices by the upmost RME and RME/DF emissions. This was not seen for the DF exhaust. In contrast, GSH was concentration-related and strongly reduced by all exhausts. This effect was most pronounced for the DF/ RME blend followed by RME and pure DF. Differences between filtered and unfiltered emissions occurred only for the blend. The blend yielded stronger toxicity when the exhaust was filtered. TNFα was two-fold elevated at 5, 10, and 15% auf the exhaust but remained unchanged above 15%. Toxicity (which was apparent from apopto-sis) at high concentrations might have diminished TNFα release. However, this release was not very pronounced anyway. DF induced a concentration-related apoptosis. The unfiltered exhaust was most effective with the exception of the upmost concentrations. RME and the blend induced no apoptosis (Morin et al., 2000). In conclusion, the strongest effects were again induced by the blend.

The study of Jalava and colleagues (2010) which is described above likewise investigated cytotoxicity, apoptosis, inflammatory effects (induction of Macrophage Inflammatory Protein 2 (MIP-2) and Tumor-Necrosis-Factor-α) and production of reactive oxygen species (ROS) in cells which were exposed to DEE from DF and RME. DEE extracts were prepared by sonication in aqueous solution and 0.3% DMSO. Concentration-related effects were seen for most markers but in contrast to previous studies no notable differences were found in regard to the different fuels, with the exception of MIP-2 and TNF-α induction which was quite weak for RME. DF caused stronger TNF-α induction whereas ROS induction was elevated by RME extracts.

### Influence of exhaust after-treatment

Regularly, exhaust after-treatment reduces significantly the toxic emissions of diesel engines. However, under certain conditions unexpected results were seen concerning mutagenic effects of the exhaust (Kado et al., 2001; Bünger et al., 2001, 2006). DEE can be treated by a diesel oxidation catalyst (DOC), a diesel particle filter (DPF) regularly combined with a DOC, or a selective catalytic reduction system (SCR). A DOC mainly reduces HC and CO of the exhaust, the DPF which is consistently combined with a DOC minimizes PM emissions, and the SCR additionally reduces NO_*x*_.

A study which was conducted by Bagley and coworkers (1998) compared SME and DF emissions concerning PM, PAH, nPAH, and toxicity. The experiments were performed with and without a DOC. Use of DOC reduced PM in DF and biodiesel exhaust by 50–80%. The DOC caused a moderate shift in the particle size/volume distribution to smaller particles and a reduction of particle volume concentrations at some of the tested conditions for both fuels. However, the solid portion of the PM was lower in SME emissions, whereas the extractable portion was slightly higher. This confirms previous studies and was attributed to an elevated generation of high molecular weight hydrocarbons when biodiesel is combusted (McDonald et al., 1995; Purcell et al., 1996). Other authors proposed that this is caused by a higher portion of unburned fuel (Bünger et al., 2000a). Particle-associated PAH and 1-nitropyrene emissions were lowered with DOC. Vapor-phase PAH emissions were reduced by DOC up to 90%. These effects resulted also in nearly 50% reduction of particle and vapor-phase-associated bacterial mutagenicity with both fuels (Bagley et al., 1998).

In a second study, Bünger et al. (2006) investigated the exhaust treatment using a DOC. It resulted in a reduced mutagenicity with and without metabolic activation at the most loads of the engine. However, direct mutagenic effects under heavy load conditions were significantly increased by use of the DOC for RME (in TA98 and TA100) and SME (only in TA98), not significantly for DF and LS-DF. The authors proposed that the DOC increases formation of direct acting nPAH by the reaction of NO_*x*_ with PAH under high load conditions leading to a high NO_*x*_ content of the exhaust and a very hot catalyst.

Exhaust after-treatment with a DPF yielded an over 95% reduction of the particle emissions (mass and numbers of all sizes) and a low mutagenicity of particle extracts for DF and RME. However, during the regeneration of the DPF a strong increase of particle emissions and muta-genicity of the extracts was observed, both exceeding the initial levels without DPF. The authors hypothesized, that high amounts of particles were released from the DPF during the regeneration phase (Bünger et al., 2004).

Krahl et al. (2006) studied the Influence of RME containing 10 ppm phosphorous (RME_10_) on the durability of a SCR system during a 1000-h endurance test. The new SCR system (DOC and SCR catalyst) decreased PM emissions to 0.01 g/kWh (Euro III limit: 0.1 g/kWh). The PM emissions increased to 0.017 g/kWh after ageing of the system. NO_*x*_ was reduced about 50%. Low NO_*x*_ reduces the formation of nPAH many of them being strong direct mutagens. In consequence, mutagenicity of PM extracts was very low compared to earlier comparable investigations without SCR, indicating a very effective reduction of mutagenic exhaust constituents confirmed by the lowest mutagenicity observed so far. Concerning mutagenic emissions the endurance test revealed an acceptable durability in particular towards phosphorous. RME_10_ had no notable effect on DEE mutagenicity.

The study of Jalava et al. (2010) included operation of the engine with a DOC/POC (no further details given). In contrast to previous studies no differences regarding the use of DOC/POC were observed for all investigated markers, with the exception of RME combustion, which yielded reduced strand breaks and ROS induction with the catalyst (note: the figures and legends of the data for strand breaks and ROS are interchanged in this publication). As stated above possible differences might have been masked because the authors referred effect markers to particle mass instead of exhaust volume (L), driven distance (km) or provided energy (KW/h).

In conclusion, after-treatment of DEE by a DOC, a DPF, or a SCR system reduces emissions independent of the combusted fuel. The effectiveness of the systems is increasing from DOC to DPF and SCR, the last leading to emissions below the Euro VI standard (valid in 2013).

### Other biogenic diesel fuels

Besides biodiesel, other fuels from renewable sources were developed and tested for their suitability in diesel engines due to various reasons. However, only few comparative investigations concerning the toxicity of their exhausts were performed up to now. Pure VO, namely rapeseed oil (RSO), was used in German truck feets and agricultural tractors due to its lower price. A stronger mutagenicity of the exhaust from the VO was observed which exceeded the mutagenicity of biodiesel and DF up to 16-fold despite similar levels of regulated emissions (Bünger et al., 2007). The strong mutagenicity mostly stopped usage of RSO as fuel along with the risk of damaging the engines. An interesting biogenic fuel is so called “biomass to liquid” (BTL). Using pyrolized biomass, the fuel is synthesized with the Fischer-Tropsch-process leading to aliphatic hydrocarbons which should result in a very clean DF according to results from the analogous fuel “gas to liquid” (GTL, from natural gas) which is already introduced to the market (Bünger et al., 2007). However, production of BTL is laborious and large scale facilities are still needed for market introduction of this fuel. Another promising biofuel for diesel engines already introduced to the market is HVO. First results show a reduction of regulated emissions (Kuronen et al., 2007, Aatola et al., 2008), a weak *in vitro* toxicity (Jalava et al., 2010), and a very low mutagenicity (Munack et al., 2010). However, Jalava et al. (2010) observed an elevation of inflammatory markers and an increase of ROS after HVO combustion. The interpretation of the results of Jalava and coworkers is hampered because data are referred to PM and the different PM emissions of HVO compared to DF were not considered.

## Conclusions

Combustion of biodiesel leads to lower emissions of PM, CO, and HC including PAH and mutagenicity in most studies, yet NO_*x*_, are regularly and aldehydes as well as cytotoxicity are often increased when compared to emissions from DF. However, recent studies show decreased PM and a very low mutagenicity of DF exhaust as well, probably caused by elimination of sulfur in present DF qualities and the use of new technology diesel engines. The use of blends with content up to 5% biodiesel has no significant impact on the emissions and their toxicity. An increased mutagenicity was observed with blends containing 20% biodiesel while other parameters changed as expected from the shares of the fuels. Combustion of pure VO in common diesel engines causes strong mutagenicity of the exhaust, while regulated emissions are low. According to preliminary results HVO seems to be a promising new biofuel emitting low emissions and causing weak toxic effects. Nevertheless, after-treatment of DEE is necessary to minimize DEE. The systems, namely DOC, DPF, and SCR, are effective independent of the combusted fuel. The use of DOC and DPF may cause paradox effects under specific working conditions. Therefore and due to its very high efficiency the SCR system seems to be the best solution until now.

No comprehensive risk assessment for DEE from biodiesel and its blends is possible up to now due to rare data from epidemiologic and animal studies. Analytical results of the regulated emissions are of suffcient consistency supporting substantial reduced hazards from PM, CO, and HC. However, the increase of NO_*x*_ may pose a hazard for enhanced irritant effects of biodiesel emissions. Data basis from measurements of non-regulated emissions is much smaller, but shows trends for lower PAH and higher aldehyde emissions from biodiesel exhaust so far. Aldehydes are irritants, but concentrations in the exhaust of biodiesel fuelled engines stay well below occupational exposure limits, indicating a negligible hazard from this exposure under normal circumstances. Lowered PAH should result in a lowered mutagenicity and this was confirmed by nearly all reviewed studies. Nevertheless, increased mutagenic effects were observed under specific conditions. Accordingly, the problem concerning blends of DF with biodiesel (B20) should be investigated with high priority.

In regard to a comprehensive hazard characterization it is urged to develop a panel of standardized and internationally accepted protocols which allow a reliable assessment of possible health hazards which may arise from the combustion of new fuels compared to conventional DF. These methods should be robust and should refflect the various health hazards associated with DEE to supplement data on regulated emissions. Methods for the generation of the exhaust and sample preparation should be harmonized.
